# Quality assessment of clinical guidelines in the care of laryngitis and pharyngitis according to AGREE II

**DOI:** 10.1590/2317-1782/e20240016en

**Published:** 2025-01-27

**Authors:** Lucas Mateus Rodrigues Carvalho, Ana Paula de Oliveira Barbosa, Nara Amanda Laismann, Débora Santos Lula Barros, Rodrigo Fonseca Lima, Rafael Santos Santana

**Affiliations:** 1 Departamento de Farmácia, Universidade de Brasília – UnB - Brasília (DF), Brasil.

**Keywords:** Laryngitis, Pharyngitis, AGREE II, Evidence-based Practice, Pharmaceutical Services, Practice Guideline

## Abstract

**Purpose:**

The study aimed to identify and assess the methodological quality of essential clinical guidelines for the management of laryngitis and pharyngitis.

**Methods:**

A systematic search of clinical guidelines for the management of laryngitis and pharyngitis was performed in three databases. Methodological quality was assessed according to AGREE II, in which each item in its domains was scored by four independent evaluators. To determine the agreement, a weighted Kappa square statistic calculation was performed.

**Results:**

81 studies were found in the bibliographic sources consulted and all were evaluated. Considering the exclusion criteria, seven guidelines were selected for final evaluation by AGREE II. The squared weighted Kappa coefficient calculated after the first round of evaluation by AGREE II was 0.85, within an almost perfect agreement rate. The domains “stakeholder engagement”, “applicability”, and “editorial independence” had the lowest mean scores and the highest standard deviation indices. They had, respectively, a mean score of (63.7%) with a standard deviation of (17%), a mean score of (65.7%) with a standard deviation of (22%), and a mean score of (35%) with a standard deviation of (23%). The use of penicillin, erythromycin, ampicillin, amoxicillin, azithromycin and clarithromycin were recommended in (75%) of all guidelines that presented pharmacological measures. As a non-pharmacological measure, oral rehydration and gargling were recommended by two guidelines.

**Conclusion:**

The statistical results indicate that all guidelines were considered as recommended. However, no uniformity was observed in the recommendations of these seven guidelines with regard to non-pharmacological and pharmacological treatment.

## INTRODUCTION

Laryngitis is the inflammation that occurs in the larynx and can cause swelling in the region of the true vocal cords. Laryngitis can be acute or chronic, infectious or non-infectious. Unlike acute laryngitis, chronic laryngitis persists over a period longer than three weeks. Pharyngitis is the inflammation of the pharynx and can be caused by viruses or bacteria. Pharyngitis has a rapid onset of sore throat and pharyngeal inflammation with or without exudate^(1-4)^.

According to a review conducted in 2010 by the Royal College of General Practitioners in the UK, an average occurrence of 6.6 cases of acute laryngitis and tracheitis per 100,000 patients per week was observed. Although the occurrence of chronic laryngitis is not yet well established, it is estimated at 3.47 diagnoses per 1000 people per year^(4)^. Regarding pharyngitis, episodes occur more in winter and the incidence of enteroviral infection has its highest cases in summer and fall. It is noteworthy that pharyngitis affects more children than adults during the winter months when group A *Streptococcus* (GAS) affects up to 20% of children. However, GAS pharyngitis occurs in less than a third compared to all cases of acute pharyngitis^(5)^.

Laryngitis and pharyngitis are considered self-limiting health problems. Thus, the correct management of these self-limiting disorders is of great importance for the public health system regarding the differential diagnoses of the various respiratory infections and the aggravation they can cause. Evidence-Based Healthcare is an approach that uses tools that guide a better outcome of scientific evidence applied in clinical practice. In order to achieve better clinical decision-making, health professionals can use evidence-based protocols and/or clinical guidelines to achieve better patient care^(6-10)^.

Clinical guidelines are informational documents with optimized recommendations for patient care. As a proposal, guidelines should be evidence-based and created based on reviews of the evidence found. Due to the fact that there is a lot of information and variability in the quality of information, it is necessary to develop guidelines in order to facilitate access to this information and recommendations based on multiple sources, thus providing greater reliability for the health professional to obtain good decision making^(11)^.

It is worth mentioning that no significant/updated data were found regarding the number of cases of Laryngitis and Pharyngitis in Brazil. Since it is difficult to collect accurate data because these cases are usually not reported^(4)^.

In Brazil, a series of already published guidelines can be found, including some guidelines that have this same methodology, however with different subjects, such as pharmaceutical care for fever^(12)^, constipation^(13)^, smoking cessation^(14)^, and headache^(15)^.

Thus, this article seeks to evaluate the methodological quality of the clinical guidelines already published regarding the management of laryngitis and pharyngitis, as well as to identify the main recommendations determined by these guidelines.

## METHODS

As this is a literature review that does not involve data collection or user participation, it was not necessary to submit the study to the ethics committee, in accordance with Resolution 466/2012 of the Health National Council.

The structure of the PICO tool was used (acronym for P: Population; I: Intervention; C: Comparison; O: Outcome). From which the clinical question was elaborated “What is the quality of the guidelines for carrying out the review of the methodological quality of the guidelines already published in the literature, as well as their main recommendations?”.

To identify clinical guidelines, a systematic search was conducted in June 2022. The search was conducted in PubMed, Virtual Health Library, and Cochrane databases. The search strategy used MeSH/DeCS descriptors with Boolean operators, resulting in: “Laryngitis” OR “Pharyngitis” AND “Guideline”. Soon after, in a second moment, a new search was performed in the PubMed database with the filter: “Guidelines” in order to find more studies focused only on guidelines.

Inclusion criteria were guidelines addressing the management of acute laryngitis, acute pharyngitis, hoarseness and sore throat, limited to the last 10 years, written in Portuguese, English, and Spanish. Exclusion criteria were guidelines addressing influenza, cold, tonsillitis, and similar conditions. In addition, the selection went through the peer review process in order to investigate for inclusion and exclusion of studies.

To assess the quality of the selected clinical guidelines, the Appraisal of Guidelines for Research & Evaluation II (AGREE II) tool was used. This tool aims to address issues related to variability in the quality of clinical guidelines, providing a set of criteria that help assess the quality of guidelines and contribute to better clinical decision-making. AGREE II evaluates 23 items organized into six domains: scope and purpose; stakeholder involvement; rigor of development; clarity of presentation; applicability; and editorial independence^(11,16,17)^.

Each item was scored by four independent assessors, previously trained and familiar with the method in question. These four people rated the guidelines with scores from 1 to 7, according to the Likert scale, according to what they agreed or disagreed with the characteristics of each guideline and whether they met the criteria determined by AGREE II users. Thus, the percentage of adequacy of each item and domain was obtained from the sum of the assigned scores and the maximum score.^(11)^

In addition, in order to have a greater comparison of the values of each domain, the arithmetic mean and standard deviation were calculated to obtain a better visualization. It is worth mentioning that the higher the score of each item, the better its quality will be^(11,18-20)^. Accepting the methodologies suggested by other authors, this article defined as “recommended” those guidelines with a score greater than 50% in the development rigor domain, and in other domains. When it is the case of having 30 to 50% in development rigor and more than 50% in two domains, it is considered “recommended, but with modifications”. When it is the case that the guideline is lower than 30% in the item “Rigor of development”, it was considered as “not recommended”^(20)^.

To determine the agreement between the four evaluators and generate greater reliability in the results, a weighted quadratic Kappa statistic calculation was performed, considering scores 1 and 2 as “low”, scores between 3 and 5 as ” intermediate” and scores 6 and 7 as “high”^(21)^. The response scale was categorized in such a way, as the evaluators decided together that scores between 1 and 2 would be as if the evaluators “completely disagreed”, scores between 3 and 5 (would be intermediate responses), and scores between 6 and 7 (completely agreed), due to the variability of responses. The Kappa coefficient was interpreted according to the degree of agreement characterized as “slight” (0 to 0.2), “reasonable (0.2 to 0.4), “moderate” (0.4 to 0.6), “substantial (0.6 to 0.8) or “almost perfect” (0.8 to 1.0) ^(21)^.

## RESULTS

**Figure 1:**
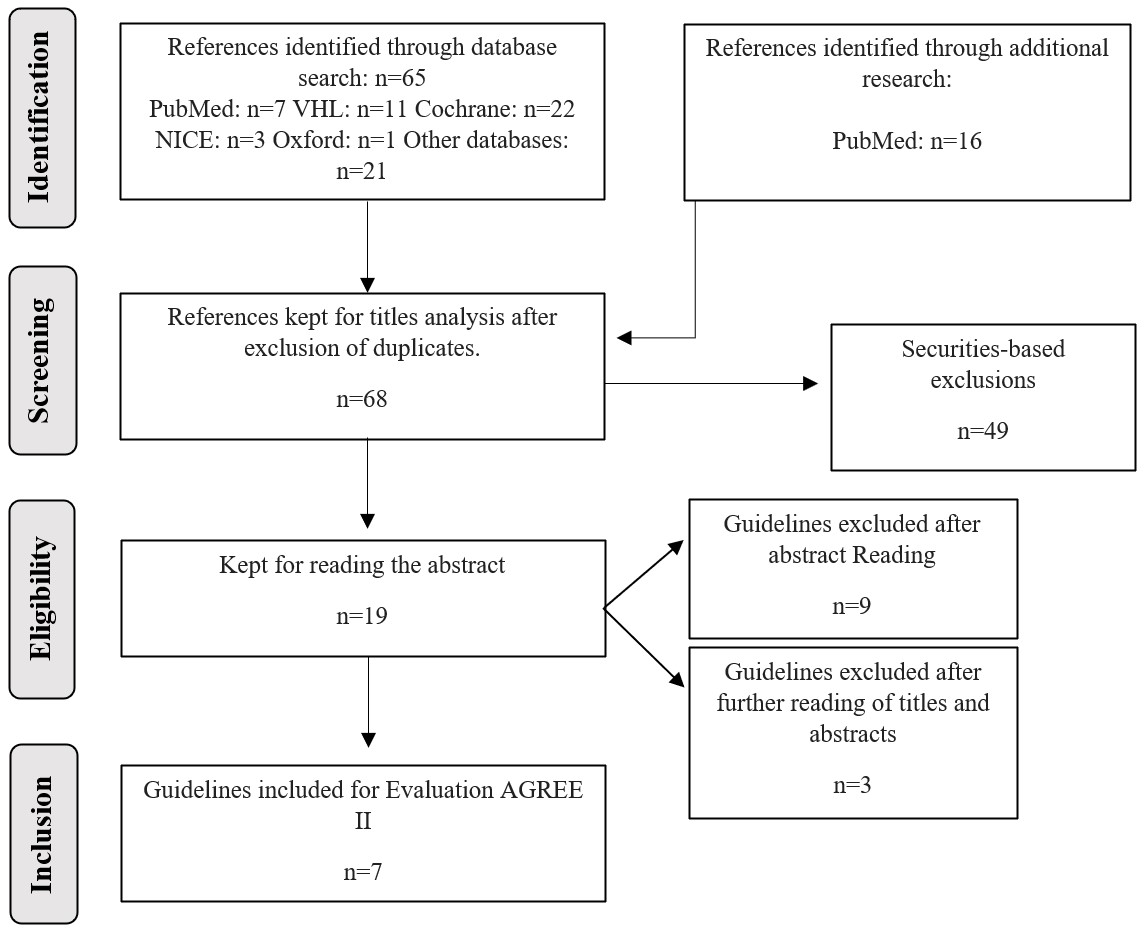
Flowchart of search and selection of existing guidelines for the management of Laryngitis and Pharyngitis.VHL: Virtual Health Library; NICE: National Institute for Health Care Excellence

When determining duplicates, 81 studies were found in the bibliographic sources consulted. Considering the exclusion criteria, based on the titles and abstracts, 7 guidelines were selected for the final assessment by AGREE II, as shown in Figure 1.

The Clinical Practice Guidelines (CPG) included for AGREE II assessment are described in Table 1, with three North American and four European guidelines as their source of origin. The squared weighted Kappa coefficient calculated after the first round of evaluation by AGREE II was 0.85, falling within a near perfect agreement rate.

**Table 1:** CPG selected for evaluation^(22-28)^

**Acronym**	**Year**	**Title (Reference)**	**Organization**	**Country**
**CPG 1**	2017	Choosing Wisely: The Top-5 Recommendations from the Italian Panel of the National Guidelines for the Management of Acute Pharyngitis in Children.	Clinical Therapeutics	United States
**CPG 2**	2012	Clinical practice guideline for the diagnosis and management of group A streptococcal pharyngitis: 2012 update by the Infectious Diseases Society of America.	Clinical Infectious Diseases	United States
**CPG 3**	2018	Clinical Practice Guideline: Hoarseness (Dysphonia).	Otolaryngology-Head and Neck Surgery	England
**CPG 4**	2015	Finnish guidelines for the treatment of laryngitis, wheezing bronchitis and bronchiolitis in children.	Acta Paediatrica	Norway
**CPG 5**	2013	Rational use of antibiotics for the management of children's respiratory tract infections in the ambulatory setting: an evidence-based consensus by the Italian Society of Preventive and Social Pediatrics.	Paediatric Respiratory Reviews	England
**CPG 6**	2019	Hoarseness Guidelines Redux: Toward Improved Treatment of Patients with Dysphonia.	Paediatric Respiratory Reviews	United States
**CPG 7**	2018	Sore throat (acute): antimicrobial prescribing	National Institute for Health and Care Excellence	England

### General guideline recommendations

In the seven guidelines evaluated, non-pharmacological therapies were not widely discussed. Only CPG's 1, 2, and 3 provided some recommendations. Eight main recommendations were identified, described in Table 2^(22-24)^.

**Table 2:** Major non-pharmacological treatment recommendations for laryngitis and pharyngitis^(22,27,28)^

**Recommendation**	**CPG 1**	**CPG 2**	**CPG 3**	**CPG 4**	**CPG 5**	**CPG 6**	**CPG 7**
**Voice rest**	-	-	Yes	-	-	-	-
**Soft sigh phonation**	-	-	Yes	-	-	-	-
**Inhalation**	-	-	Yes	-	-	-	-
**Oral rehydration**	Yes	-	Yes	-	-	-	-
**Reduction of caffeinated drinks**	-	-	Yes	-	-	-	-
**Avoid using tobacco**	-	-	Yes	-	-	-	-
**Avoid being in closed places**	-	-	No	-	-	-	-
**Gargle with warm water**	-	Yes	Yes*	-	-	-	-

* Just gargle

**Caption**: (-) No non-pharmacological treatments were addressed in CPG

In non-pharmacological measures, oral rehydration, as well as gargling, is the most recommended (66.66%), followed by voice rest (33.33%), as well as other non-pharmacological treatments recommended by the third guideline.

Recommendations for the pharmacological treatment approach were explicitly mentioned by framework guidelines (CPG 1, CPG 2, CPG 5, and CPG 7). Three guidelines did not address any pharmacological treatment measures (CPG 3, CPG 4 e CPG 6), shown in Table 3. Considering the recommendations cited, penicillin, erythromycin, ampicillin/sulbactam, amoxicillin, azithromycin, and clarithromycin were recommended in 75% of all guidelines. Cephalexin, clindamycin, ibuprofen, and paracetamol were recommended in 50% of all guidelines, followed by cefadroxil, corresponding to 25% of guidelines^(22,23,26,28)^.

**Table 3:** Pharmacological treatment recommendations^(22,27,28)^

**Medicinal products**	**CPG 1**	**CPG 2**	**CPG 3**	**CPG 4**	**CPG 5**	**CPG 6**	**CPG 7**
**Ampicillin/ Sulbactam**	Yes	Yes	-	-	Yes	-	No
**Amoxicillin**	Yes	Yes	-	-	Yes	-	No
**Azithromycin**	Yes	Yes	-	-	Yes	-	No
**Cefadroxil**	No	Yes	-	-	No	-	No
**Cephalexin**	No	Yes	-	-	Yes	-	No
**Clarithromycin**	No	Yes	-	-	Yes	-	Yes
**Clindamycin**	Yes	Yes	-	-	No	-	No
**Erythromycin**	No	Yes	-	-	Yes	-	Yes
**Ibuprofen**	Yes	Yes	-	-	No	-	No
**Paracetamol**	Yes	Yes	-	-	No	-	No
**Penicillin**	Yes	Yes	-	-	Yes	-	No

**Caption:** (-) No pharmacological treatment was discussed

### Assessment of the quality of the guidelines according to AGREE II

The percentage for each domain of the clinical guidelines is presented in Table 4. When applying the average to the selected guidelines, all were considered as recommended to be used by health professionals, attesting to the quality of development.

**Table 4:** Percentage of adequacy in the quality assessment domains of the AGREE II

**Domain**	**CPG 1**	**CPG 2**	**CPG 3**	**CPG 4**	**CPG 5**	**CPG 6**	**CPG 7**	**Average**	**Standard deviation**
**D1**	100	100	100	69	100	100	100	95.6	12
**D2**	43	71.4	83.3	51.2	86	51.2	59.5	63.7	17
**D3**	62	92	91	69.2	80.4	92	91	82.6	12
**D4**	100	100	100	92	100	100	100	98.9	3
**D5**	46.4	53	60	37	79.5	96.4	87.5	65.7	22
**D6**	14.3	57.1	71.4	14.3	14.3	46.4	27	35	23

**Caption:** D1: scope and purpose; D2: stakeholder involvement; D3: rigor of development; D4: clarity of presentation; D5: applicability; D6: editorial independence

The domain with the highest mean score was clarity of presentation (D4), with 98,9% adequacy. This domain had no CPG with scores below 90%. It also had the lowest weighted standard deviation (3%). The second domain with the highest mean score was scope and purpose (D1), with 95.6% adequacy. This domain had all CPG scores above 60% and a standard deviation of 12%. The third domain with the highest mean score was rigor of development (D3), with 82.6% adequacy. This domain has a standard deviation of 12%.

The three domains with the lowest mean scores as well as the highest standard deviations analyzed were stakeholder engagement (D2), applicability (D5), and editorial independence (D6). They had, respectively, a mean score of 63.7% with a standard deviation of 17%, a mean score of 65.7% with a standard deviation of 22% and a mean score of 35% with a standard deviation of 23%. Still regarding these domains, scores above 50% were achieved in D2 and D5 by six (CPG 2 = 71.4%; CPG 3 = 83.3%; CPG 4 = 51.2%; CPG 5 = 86%; CPG 6 = 51.2%; CPG 7 = 59.5%) and five (CPG 2 = 53%; CPG 3 = 60%; CPG 5 = 79.5%, CPG 6 = 96.4%; CPG 7 = 87.5%), respectively, but only two guidelines in D6 achieved scores within this range (CPG 2= 57.1%; CPG 3= 71.4%).

The CPG with the highest scores was CPG 3, with all scores except one (D5= 60%) above 70%. The CPG with the lowest score was CPG 4 due to the low percentage of D5 = 37% and D6 = 14.3%. The maximum possible score (100%) was assigned to six guidelines (CPG 1, CPG 2, CPG 3, CPG 5, CPG 6 and CPG 7) in D1 and D4. In addition, scores below 20% were highlighted for CPG 1, CPG 4 and CPG 5 (D6 = 14.3%).

## DISCUSSION

According to the criteria adopted, a cut-off point of 50% was determined, in which all the guidelines of this study were considered as recommended. In view of the studies that determined a cut-off point, the rigor of the development domain (D3) may be a stronger quality indicator when compared to the other domains of the instrument, being able to argue that the guidelines show improvement in their presentation, indicates minimal bias and the development of evidence-based guidelines, but that there is still room for improvement in the methods and in other quality domains^(29,30)^.

If we consider the cut-off point of 50% in D3 as the only criterion judged, all guidelines would be considered high quality. However, if the cut-off point of 50% at D3, D5, and D6 are considered, only two guidelines (CPG 2 = 92%, 53%, and 57.1%; CPG 3 = 91%, 60%, and 71.4%) assessed by this article would be considered high quality, both of which cite non-pharmacological management by gargling with water as a treatment^(23,24)^.

Studies show that there is a low level of recommendation for throat preparations containing local anesthetics and/or non-steroidal anti-inflammatory drugs^(31,32)^. Therefore, rest, adequate fluid intake and avoidance of irritants are recommended. Management of laryngitis and pharyngitis depends on the severity, with most conditions being self-limiting. One of the treatment options includes vocal hygiene, referring to measures such as voice rest, hydration, humidification, and limiting caffeine intake^(33)^. Among the seven guidelines studied, CPG 3 had the highest scores regarding AGREE II scores, and it was also the only one that presented all the general recommendations cited, except for staying indoors^(24)^. However, this demonstration of quality does not mean that the other guidelines are of lesser quality than CPG 3.

Clinical guidelines gain an important place in the practice of health professionals, highlighting the importance of well-founded guidelines in bridging gaps between research and clinical practice^(34)^. The scarcity of non-pharmacological interventions in most guidelines demonstrates a lack of criteria in the selection of evidence for the construction of these guidelines. Non-pharmacological treatment is an alternative approach to drug treatment and can be effective in relieving symptoms and providing recovery. Despite the prevalence of laryngitis and pharyngitis in the population, treatment is far from clear, demonstrating variability between existing clinical guidelines^(35)^.

Regarding the recommendations for pharmacological treatment, CPG 2 stood out, since of the 17 drugs cited, 11 were recommended in this guideline^(23)^. It is also the third guideline with the highest mean score across all domains. The guideline addresses that, with some exceptions, antimicrobial therapy does not have many proven benefits for the treatment of acute pharyngitis caused by microorganisms other the *streptococcus A* virus. A Cochrane review cites a study that investigated the use of erythromycin for acute laryngitis, in over 100 patients, and demonstrated that it appears to be effective in reducing vocal disturbances as measured by participants after one week and cough after two weeks. However, the quality of evidence was very low for all outcomes, highlighting that some antibiotics, such as erythromycin, do not appear to be effective in treating acute laryngitis when assessing objective outcomes^(36)^.

In CPG 1 and 5, 7 drugs were recommended in each guideline^(22,26)^. CPG 7 presented 1 recommended medicine^(28)^, while CPGs 3, 4, and 6 did not present any of these drugs as a pharmacological treatment measure. Thus, it is concluded that no uniformity was observed in the recommendations of these seven guidelines regarding non-pharmacological and pharmacological treatment.

Of the over-the-counter medicines in Brazil, only ibuprofen and paracetamol were cited. Ibuprofen can be offered for the short-term symptomatic treatment of sore throat, slightly more effective than paracetamol, encompassing greater experience in pediatrics.^(31)^ Antibiotic treatment was mentioned in most guidelines, with the aim of shortening the time of illness, eradicating the bacteria and avoiding contagion. However, it is understood that antibiotics should not be administered unless microbiological confirmation of streptococcal infection has been performed^(32,37)^. According to the spectrum of activity, occasional adverse reactions and costs, penicillin or amoxicillin are recommended drugs of choice for those who are not allergic^(38-40)^.

The AGREE II tool focuses on assessing the quality and methodological rigor of the entire CPG process and cannot assess the effectiveness of individual recommendations^(41)^. AGREE II does not assess the content of the guideline, but assesses the methods used in the development and quality of the reports. It is not explicit how the different domain scores should be weighted to differentiate guidelines classified as high or low quality, but recommends that this decision should be made by the user in the specific context^(42,43)^.

In general, treatment is directed toward symptom control with voice rest, humidification, and analgesic therapy. In pharmacological recommendations, there are public health consequences through antibiotic stewardship and the high rates of bacterial resistance. Studies cite that microbiological testing should not be performed regularly because antibiotics have a limited effect in shortening the clinical course and should be reserved for well-selected cases^(36,40,44)^.

In this study, the three domains that most influenced the quality of the guidelines were those with the lowest mean scores and the highest standard deviations. This indicates that the scores of these domains could be further improved by providing more specific information regarding the inclusion of individuals about their opinions and experiences (D2), the development and implementation of the guideline (D5), and the lack of information about funding sources and conflict of interest (D6)^(29)^.

The guideline with the lowest mean score (CPG 4) had the lowest domains of applicability (D5), and transparent editorial (D6) of all the guidelines studied. This can be explained by the lack of information on dissemination and implementation strategies or even the absence of monitoring criteria. Another transparent point is the lack or insufficiency of information on the presence of conflicts of interest, as institutions should be transparent about their conflict of interest policies and funding sources^(29)^.

As analyzed, the guidelines were produced in developed countries, which may be influenced by the greater involvement and funding of public institutions with more guidelines developed by specialized societies. All guidelines had benefits either in recommendations for management or a more accurate diagnosis of laryngitis and pharyngitis. Some limitations of this study include restricted access and unindexed guidelines in some databases. Therefore, the guiding question was answered, given that the methodological quality of the seven selected guidelines was assessed, where relevant information can be brought about their main recommendations.

It is worth highlighting the importance of clinical guidelines, as they are informative documents designed to optimize the treatment offered to the patient. The guidelines are scientifically based, taking into account the risk-benefit of different health care options. Thus, due to the large volume of information as well as its variability in the quality of the information, there is a need to develop clinical practice guidelines facilitating access to this information, as well as its recommendations^(45)^. In this way, there is an improvement in the health process and planning with guidelines for these topics, thus benefiting an advance in the quality of care.

In relation to Brazilian Clinical Practice, there is variability of information mainly on Brazilian websites, with no scientific studies found that significantly address the treatment of Laryngitis and Pharyngitis. However, due to the lack of precision and confidence in the results of Brazilian clinical practice, it was decided not to include them in the study. The scarcity of scientific studies on how they are carried out or how they can be carried out in Brazilian clinical practice on these self-limited diseases is, without a doubt, a worrying issue, especially given the abundance of studies carried out outside Brazil. It is worth mentioning that a study proposed by Santos et al. highlights the challenges in health research in Brazil, with some concerns such as undergraduate students who may not have support to dedicate themselves to scientific academic development, lack of mobilization to promote financial increase in research and development^(46)^. These are some of the concerns regarding health research in Brazil. Therefore, it is important to highlight that limitations in understanding how these practices are carried out may restrict a comprehensive view of Laryngitis and Pharyngitis. We can infer, therefore, that there are challenges in accessing important information in the Brazilian context, but even with the challenges that may be encountered during research, there is the possibility of applying the recommendations in the Brazilian context, however there is a need to prepare clinical practice documents. As was done in the pharmaceutical care guidelines for fever^(12)^, cold^(13)^, smoking cessation^(14)^ and headache^(15)^.

## CONCLUSION

Of the seven guidelines analyzed, the majority incorporated drug treatment strategies, while less than half considered non-pharmacological approaches. The statistical results demonstrate that all guidelines were considered as recommended. However, the findings highlight the variability of recommendations for the management of laryngitis and pharyngitis between guidelines. The results of this analysis highlight the importance of improving the process of planning clinical guidelines for these topics, which tends to represent an advance in the quality of care.

## References

[1] Ada Health GmbH (2022). Acute pharyngitis: symptoms: viral: bacterial.

[2] Donowitz JR (2023). Acute pharyngitis: symptoms, diagnosis and treatment.

[3] ABORL CCF: Associação Brasileira de Otorrinolaringologia e Cirurgia Cérvico-Facial (2019). Guideline IVAS: infecções das vias aéreas superiores.

[4] Whited CW, Dailey SH (2023). Laryngitis: symptoms, diagnosis and treatment.

[5] Donowitz JR (2023). Acute pharyngitis: epidemiology.

[6] Galvao CM, Sawada NO, Rossi LA (2002). Evidence-based practice: theoretical considerations for its implementation in perioperative nursing. Rev Lat Am Enfermagem.

[7] Brasil (2016). Methodological guidelines: elaborating clinical.

[8] Shaneyfelt TM, Mayo-Smith MF, Rothwangl J (1999). Are guidelines following guidelines?. JAMA.

[9] Djulbegovic B, Guyatt GH (2017). Progress in evidence-based medicine: a quarter century on. Lancet.

[10] São Paulo (2022). Saúde Baseada em Evidências (SBE).

[11] AGREE Enterprise (2013). Advancing the science of practice guidelines.

[12] Costa AP, Reis TM, Santana RS (2022). Elaboração de diretriz clínica para o cuidado farmacêutico na febre. Int J Dev Res.

[13] Lima BFR, Alves BMCS, Tavares NUL, Lima RF, Ginani VC, Reis TM (2022). Quality appraisal of clinical guidelines for the management of constipation according to AGREE II instrument. Res Soc Dev.

[14] Costa AP, Alves BMCS, Silva DLM, Martins RLM, Silva FA, Zimmermann IR (2022). Diretrizes clínicas para cessação do tabagismo: análise comparativa com o AGREE II. Brasília Med.

[15] Vaz JM, Alves BM, Duarte DB, Marques LA, Santana RS (2022). Quality appraisal of existing guidelines for the management of headache disorders by the AGREE II’s method. Cephalalgia.

[16] Brouwers MC, Kho ME, Browman GP, Burgers JS, Cluzeau F, Feder G (2010). AGREE II: advancing guideline development, reporting and evaluation in health care. CMAJ.

[17] Latorraca CD, Pacheco RL, Martimbianco AL, Pachito DV, Riera R (2018). AGREE II: a tool to assess the quality and reporting of guidelines: descriptive study. Diagn Treat..

[18] Hoffmann-Eßer W, Siering U, Neugebauer EAM, Brockhaus AC, Lampert U, Eikermann M (2017). Guideline appraisal with AGREE II: systematic review of the current evidence on how users handle the 2 overall assessments. PLoS One.

[19] Anwer MA, Al‐Fahed OB, Arif SI, Amer YS, Titi MA, Al‐Rukban MO (2018). Quality assessment of recent evidence‐based clinical practice guidelines for management of type 2 diabetes mellitus in adults using the AGREE II instrument. J Eval Clin Pract.

[20] Santana RS, de Oliveira Lupatini E, Zanghelini F, de March Ronsoni R, Rech N, Leite SN (2018). The different clinical guideline standards in Brazil: high cost treatment diseases versus poverty-related diseases. PLoS One.

[21] Kraemer HC (2015). Kappa coefficient..

[22] Chiappini E, Bortone B, Di Mauro G, Esposito S, Galli L, Landi M (2017). Choosing wisely: the top-5 recommendations from the Italian panel of the national guidelines for the management of acute pharyngitis in children. Clin Ther.

[23] Shulman ST, Bisno AL, Clegg HW, Gerber MA, Kaplan EL, Lee G (2012). Executive summary: clinical practice guideline for the diagnosis and management of group a streptococcal pharyngitis: 2012 update by the infectious diseases society of America. Clin Infect Dis.

[24] Stachler RJ, Francis DO, Schwartz SR, Damask CC, Digoy GP, Krouse HJ (2018). Clinical practice guideline: hoarseness (Dysphonia) (Update). Otolaryngol Head Neck Surg.

[25] Tapiainen T, Aittoniemi J, Immonen J, Jylkkä H, Meinander T, Nuolivirta K (2016). Finnish guidelines for the treatment of laryngitis, wheezing bronchitis and bronchiolitis in children. Acta Paediatr.

[26] Chiappini E, Mazzantini R, Bruzzese E, Capuano A, Colombo M, Cricelli C (2014). Rational use of antibiotics for the management of children’s respiratory tract infections in the ambulatory setting: an evidence-based consensus by the Italian Society of Preventive and Social Pediatrics. Paediatr Respir Rev.

[27] Francis DO, Smith LJ (2019). Hoarseness guidelines redux: toward improved treatment of patients with dysphonia. Otolaryngol Clin North Am.

[28] National Institute for Health and Care Excellence (2018). Sore throat (acute): antimicrobial prescribing.

[29] Alonso-Coello P, Irfan A, Sola I, Gich I, Delgado-Noguera M, Rigau D (2010). The quality of clinical practice guidelines over the last two decades: a systematic review of guideline appraisal studies. Qual Saf Health Care.

[30] Hoffmann-Eßer W, Siering U, Neugebauer EAM, Lampert U, Eikermann M (2018). Systematic review of current guideline appraisals performed with the Appraisal of Guidelines for Research & Evaluation II instrument: a third of AGREE II users apply a cut-off for guideline quality. J Clin Epidemiol.

[31] Krüger K, Töpfner N, Berner R, Windfuhr J, Oltrogge JH (2021). Sore Throat. Dtsch Arztebl Int.

[32] Cots JM, Alós JI, Bárcena M, Boleda X, Cañada JL, Gómez N (2015). Recomendaciones para el manejo de la faringoamigdalitis aguda del adulto. Acta Otorrinolaringol Esp.

[33] Wood JM, Athanasiadis T, Allen J (2014). Laryngitis. BMJ.

[34] Costa AP, Alves BMCS, Silva DLM, Melo Martins RL, Silva FA, Zimmermann IR (2022). Diretrizes clínicas para cessação do tabagismo: análise comparativa com o agree II. Brasília Med..

[35] Llor C, Cots JM (2015). Certezas y dudas sobre el manejo de la faringitis aguda. Aten Primaria.

[36] Reveiz L, Cardona AF (2015). Antibiotics for acute laryngitis in adults. Cochrane Database Syst Rev.

[37] Chiappini E, Bortone B, Di Mauro G, Esposito S, Galli L, Landi M (2017). Choosing Wisely: the Top-5 recommendations from the italian panel of the national guidelines for the management of acute pharyngitis in children. Clin Ther.

[38] Shulman ST, Bisno AL, Clegg HW, Gerber MA, Kaplan EL, Lee G (2012). Clinical practice guideline for the diagnosis and management of group a streptococcal pharyngitis: 2012 Update by the Infectious Diseases Society of America. Clin Infect Dis.

[39] Robinson JL (2021). Paediatrics: how to manage pharyngitis in an era of increasing antimicrobial resistance. Drugs Context.

[40] Chiappini E, Regoli M, Bonsignori F, Sollai S, Parretti A, Galli L (2011). Analysis of different recommendations from international guidelines for the management of acute pharyngitis in adults and children. Clin Ther.

[41] Chorath K, Prasad A, Luu N, Go B, Moreira A, Rajasekaran K (2022). Critical review of clinical practice guidelines for evaluation of neck mass in adults. Rev Bras Otorrinolaringol (Engl Ed).

[42] Rohde A, Worrall L, Le Dorze G (2013). Systematic review of the quality of clinical guidelines for aphasia in stroke management. J Eval Clin Pract.

[43] Vaz JM, Alves BM, Duarte DB, Marques LA, Santana RS (2022). Quality appraisal of existing guidelines for the management of headache disorders by the AGREE II’s method. Cephalalgia.

[44] Matthys J, De Meyere M, van Driel ML, De Sutter A (2007). Differences among international pharyngitis guidelines: not just academic. Ann Fam Med.

[45] Brasil (2023). Diretrizes metodológicas: elaboração de diretrizes clínicas.

[46] Santos AO, Barros FPC, Delduque MC (2019). A pesquisa em saúde no Brasil: desafios a enfrentar. Saúde Debate.

